# Er Miao San, a traditional Chinese herbal formula, attenuates complete Freund’s adjuvant-induced arthritis in rats by regulating Th17/Treg cells

**DOI:** 10.1080/13880209.2020.1720745

**Published:** 2020-02-08

**Authors:** Xing Dai, Dongping Yang, Jinping Bao, Qiying Zhang, Jiemin Ding, Min Liu, Meihuizi Ding, Mengli Liu, Juan Liang, Xiaoyi Jia

**Affiliations:** a School of Pharmacy, Anhui University of Chinese Medicine, Hefei, China; b The First Clinical Medical College, Anhui Medical University, Hefei, China

**Keywords:** Rheumatoid arthritis, inflammatory cytokine, traditional Chinese medicine

## Abstract

**Context:**

Er Miao San (EMS) is a traditional Chinese medicine composed of *Atractylodis Rhizoma* and *Phellodendri Cortex* in a 1:1 weight ratio. EMS has been used to treat rheumatism in China for many years.

**Objective:**

To evaluate the anti-arthritic activity of EMS extract on adjuvant-induced arthritis (AA) in Sprague-Dawley rats and to clarify its mechanisms of action.

**Materials and methods:**

EMS (0.75, 1.5 and 3 g/kg, once daily) was orally administered from day 18 after immunization to day 31. The effects of EMS on AA rats were evaluated by histopathological examination, paw swelling and polyarthritis index. The proliferation of fibroblast-like synoviocyte (FLS) and T cells was detected by CCK-8. The percentages of Th17 cells and Treg cells in splenocytes were determined by flow cytometry. Levels of cytokines in serum were detected by ELISA.

**Results:**

EMS treatment significantly decreased the paw volume (from 1.20 to 0.81), polyarthritis index (from 9.56 to 4.46) and alleviated ankle joint histopathology in AA rats. EMS inhibited the proliferation of FLS and T cells. Furthermore, EMS treatment decreased Th17 cells (from 4.62 to 2.08%) and increased Treg cells (from 2.77 to 4.75%) in splenocytes. The levels of IL-17A, TNF-α and IL-6 were remarkably decreased in the serum of EMS-treated rats, whereas the levels of IL-10 and TGF-β1 were significantly increased.

**Conclusions:**

EMS exhibits anti-arthritic activity in the AA model by regulating the balance of cytokines and the ratio of Th17 and Treg cells. These insights may provide an experimental basis for the clinical treatment of RA.

## Introduction

Rheumatoid arthritis (RA) is a chronic systemic inflammatory disease that is characterized by synovial hyperplasia, inflammatory cell infiltration and pannus formation in synovial tissues. Most RA patients also experience bone and cartilage destruction (Chen et al. 2019). RA has a global incidence of ∼0.8% and the health and quality of life of those afflicted is severely affected (Karami et al. 2019). The pathogenesis of RA is complex and unclear, and so is its aetiology. According to Western medicine, several different factors are involved in its pathogenesis, including genetic, infection and disorders of the immune system (Karami et al. 2019; Zamanpoor 2019). In traditional Chinese medicine, RA is called ‘Bi Zheng’, a syndrome characterized by pain; numbness of the limbs, joints and muscle; weakness of flexion and extension; and, movement disorder. Bi Zheng is caused by wind, cold, dampness, heat, amongst other processes (Guo et al. 2015). The main objective of treatment is to relieve joint pain and prevent joint damage, in order to improve a patient’s quality of life. Currently, drugs used in the clinical treatment of RA include steroidal anti-inflammatories, disease-modifying antirheumatic drugs (DMARDs), non-steroidal anti-inflammatory drugs (NSAIDs) and biological preparations (Abbasi et al. 2019). However, most of these drugs act slowly or produce severe adverse reactions after long-term application (Ma et al. 2019). Therefore, the identification of a natural drug formula with good curative effect and low adverse reactions would be a significant breakthrough.

The herbal formula, Er Miao San (EMS), is a commonly used traditional Chinese medicine. It consists of equal amounts of *Atractylodis Rhizoma* and *Phellodendri Cortex*, and was named by Zhu Danxi during the Yuan dynasty (Dan-Xi-Xin-Fa in Danxi’s Experiences in Medicine). Phellodendri Cortex is the dry bark of *Phellodendron chinense* Schneid (Rutaceae). Atractylodis Rhizoma is the dry rhizome of *Atractylodes lancea* (Thunb.) DC. (Compositae). The two herbs are named ‘Huang Bai’ and ‘Cang Zu’ in Chinese, respectively. EMS can clear heat and eliminate dampness, and this formulation has long been used in traditional Chinese medicine for the treatment of conditions such as dampness, heat, swelling and pain in the knee, lower limb erysipelas, leucorrhoea and scrotum wet itching. EMS has also been studied for its anti-hyperuricaemia effect as it reduces the level of serum uric acid and suppresses the activities of xanthine dehydrogenase and xanthine oxidase (Kong et al. 2004). A recent study demonstrated that EMS ameliorated renal impairment and hyperuricaemia in rats synergistically by upregulating organic anion transporters 1 (OAT1) and 3 (OAT3) (Guo et al. 2015). Furthermore, EMS inhibited the inflammatory mediators in RAW264.7 cells stimulated by LPS via the NK-κB pathway (Chen et al. 2014). Nevertheless, no systematic study has been performed to evaluate the potential beneficial effects and molecular mechanisms of EMS in RA. Many studies have shown that Th17 cells and Treg cells exert an antagonistic role in the initiation and progression of RA (McGovern et al. 2012; Kikodze et al. 2016; Liu et al. 2017). However, the effects of EMS on T cells have not been investigated. In this study, we explored the anti-arthritic effects of EMS on adjuvant-induced arthritis (AA) in rats, and its potential mechanisms, to provide an experimental basis for the clinical treatment of RA.

## Materials and methods

### Plant materials

The medicinal herbs, *Atractylodis Rhizoma* (1902120322) and *Phellodendri Cortex* (1901200062), were obtained from BoZou (Anhui, China) in January 2019, and authenticated by Dr. Liu SJ (School of Pharmacy, Anhui University of Chinese Medicine). A certified specimen of each sample (ID: EMS-19-01) was deposited in the Medicine specimen room, School of Pharmacy, Anhui University of Chinese Medicine (Hefei, China).

### Animals

Sprague-Dawley (SD) rats (male, 150–180 g) were purchased from the animal department of Anhui Medical University (Hefei, China). All rats were housed in a dedicated animal room and maintained at constant temperature (22 ± 5 °C) and humidity (55%+5%). Rats were randomly assigned to normal, model, EMS (0.75, 1.5 and 3 g/kg) and methotrexate (MTX) groups. The experimental protocol used in this study was conducted in accordance with the Experimental Animal Ethics Committee of Anhui University of Chinese Medicine (no.: 20190219).

### Preparation of EMS aqueous extracts

The materials of EMS were extracted three times with 10 volumes of distilled water (v/w) at 100 °C (1 h per extraction). The resulting suspension was separated by filtration, lyophilized to yield a powder, and then stored at 4 °C before use.

### Reagents

MTX was obtained from Xinyi Medical Limited Company (Shanghai, China). FITC-CD4, PE-IL-17A, APC-CD25 and PE-Foxp3 were purchased from eBioscience (San Diego, CA). ELISA kits for TNF-α, IL-6, IL-17A, IL-10 and TGF-β1 were from 4A Biotech Co., Ltd. (Beijing, China).

### UPLC analysis of EMS

EMS and the two reference substances, berberine and atractylodin, were dissolved in methanol at the appropriate concentration. UPLC was performed using a UHPLC UltiMate 3000 (Thermo, Waltham, MA). The samples were analysed on a discovery-C18 analytical column (2.1 mm × 100 mm, 1.7 μm particle size, Supelco, Bellefonte, PA). The samples were run using acetonitrile as mobile phase A, and 0.1% formic acid in water as mobile phase B. The gradient programme was used as follows: 0–5 min, 10–60% A, 5–10 min, 60–85% A, 10–15 min, 85–90% A and 15–20 min, 90% A. Chromatography analysis was performed at a flow rate of 0.2 mL/min and at room temperature (30 °C). The detection wavelength was set at 340 nm and the injection volume was 2 μL. The experimental samples and the control samples were both analysed using the same conditions.

### Induction of AA and treatment

Complete Freund’s adjuvant (CFA, 10 mg/mL) was prepared by suspending heat-killed *Mycobacterium butyricum* in sterile liquid paraffin. The AA model was induced in rats by subcutaneous injection of 100 µL of CFA into the left hind metatarsal footpad (Jia et al. 2019). Normal rats were injected with the same amount of physiological saline. After the onset of AA (around day 17), the degree of inflammation was scored and induced rats were divided into five groups randomly: model group, EMS (0.75, 1.5 and 3 g/kg) and MTX (0.5 mg/kg). EMS (once daily) and MTX (once every three days) were administered via gavage from day 18 after immunization to day 31. The normal and model groups were administered an equal volume of carboxymethyl cellulose.

### Evaluation of arthritis

Arthritis severity was evaluated by examining changes in body weight, volume of hind paw swelling and polyarthritis index. The body weight of the rats was weighed with an electronic scale every seven days. Hind paw volume was measured with a PV-200 volume metre (Chengdu Technology Market Co. Ltd., Chengdu, China) before (base value) and after immunization (days 14, 17, 20, 23, 26, 29 and 32). The polyarthritis index was scored by erythema and joint swelling ranging from 0 to 4, and a maximum arthritic score was set at 16 per animal, as discussed in an earlier study (Wu et al. 2016).

### Measurement of cytokine concentration in serum

The concentrations of cytokines in serum were measured using ELISA kits. The absorbance of each well was recorded at 450 nm using a Multiskan Spectrum (Thermo Scientific, Waltham, MA).

### Histological examination

The right ankle joints of rats were fixed in 4% paraformaldehyde, decalcified in 10% ethylene diamine tetraacetic acid, and then embedded in paraffin. Paraffin sections were stained with haematoxylin and eosin (H&E) and examined microscopically. The severity of arthritis was scored under blinded conditions from 0 to 4 according to synovium hyperplasia, mononuclear cell infiltration, pannus formation, and erosion of articular cartilage and subchondral bone as described previously (Wu et al. 2016).

### Spleen lymphocyte and fibroblast-like synoviocyte (FLS) proliferation assay

Splenocytes were isolated from spleen and suspended in RPMI-1640 with 10% foetal calf serum (FCS) at a concentration of 1 × 10^7^ cell/mL and then placed in 96-well plates at a final concentration of 1 × 10^6^ cell/mL with ConA (5 mg/L). The cultures were incubated in a carbon dioxide incubator for 72 h. CCK-8 (20 µL) was added to each well and the plates were incubated for 2 h before termination of the reactions. Absorbance was measured at 450 nm. The results are presented as the average of triplicate counts.

FLSs were isolated from synovial tissues of the knees joints and cultured in DMEM supplemented with 20% FCS in a carbon dioxide incubator as described previously (Wu et al. 2016). FLSs were resuspended and placed in 96-well plates (1 × 10^5^ cell/mL), then incubated in a carbon dioxide incubator for 48 h before CCK-8 measurement.

### Flow cytometry

To quantify the number of Treg cells, lymphocytes were first stained with CD4 (FITC) and CD25 (APC) antibodies, then subsequently fixed, permeabilized and stained with Foxp3 (PE) antibody. To quantify the number of Th17 cells, lymphocytes were first stained with CD4 (FITC) antibodies. The cells were then incubated for 8 h with a cell stimulation cocktail, and subsequently fixed, permeabilized and stained with IL-17 (PE) antibody. The stained cells were detected by flow cytometer (FC500, Beckman, Brea, CA), and the data were analysed with Flowjo (version 7.6).

### Statistical analysis

Data are shown as mean ± SD. One-way analysis of variance (ANOVA) and *post hoc* Tukey’s test were performed between multiple groups. *p* < 0.05 was considered statistically significant.

## Results

### Phytochemical analyses EMS by UPLC

**Figure 1.**
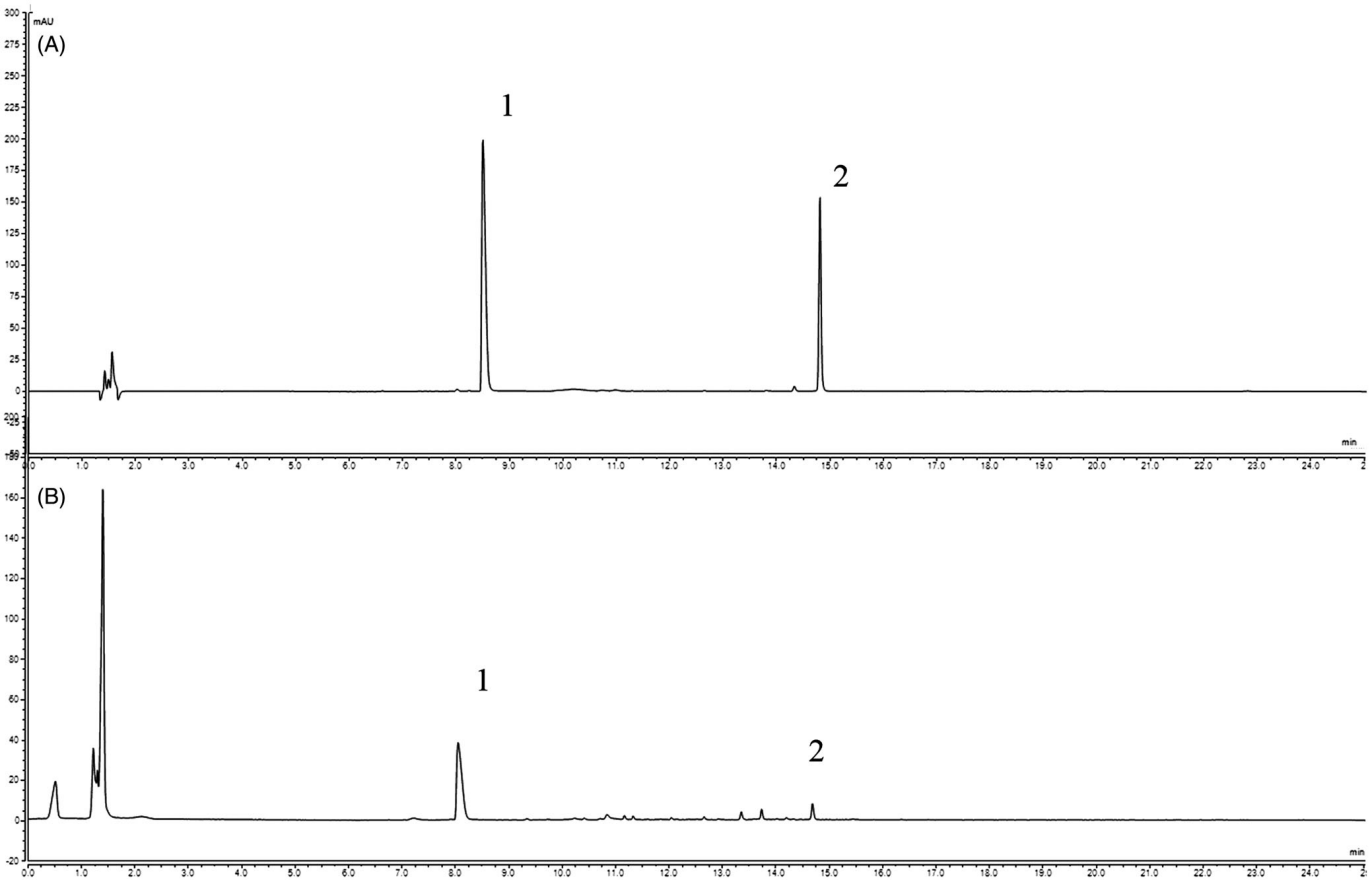
Phytochemical analyses of EMS by UPLC. (A) Reference substances; (B) EMS: **1**, berberine, **2**, atractylodin.

According to the Chinese Pharmacopoeia, berberine and atractylodin can be used as standards to investigate the quality of *Phellodendri cortex* and *Atractylodis rhizoma*, respectively. Therefore, these two compounds were used as references to verify the composition of EMS. Figure 1 shows UPLC chromatograms of the two reference compounds berberine (**1**) and atractylodin (**2**). The peaks of berberine and atractylodin in EMS were identified by comparing peak retention times with those of the reference compounds. The UPLC results confirm that EMS is comprised of berberine and atractylodin.

### EMS improved the clinical signs of AA rats

**Figure 2.**
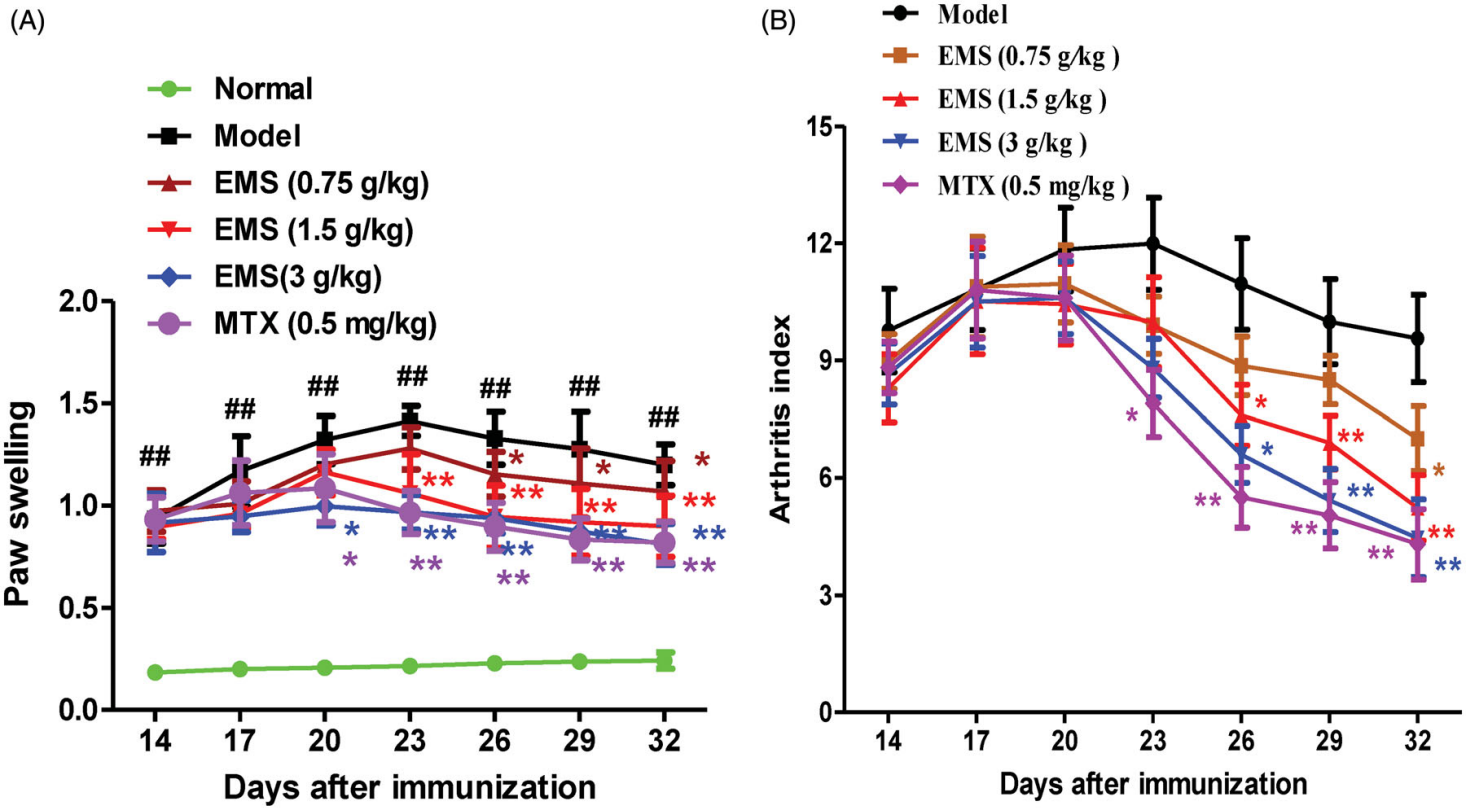
Effect of EMS on general indicators in AA rats. (A) Effects of EMS on degree of paw swelling in AA rats. ^##^*p*< 0.01 vs. normal *p< 0.05, **p< 0.01 vs. model (mean ± SD, *n* = 8). (B) Effects of EMS on polyarthritis index in AA rats. **p< 0.01 vs. model (mean ± SD, *n* = 8).

The effects of EMS were determined using an *in vivo* AA model. Following CFA injection of the rats, significant increases in polyarthritis index and paw swelling were observed. EMS (0.75, 1.5 and 3 g/kg) and MTX (0.5 mg/kg) treatment alleviated paw swelling and significantly decreased the polyarthritis index (Figure 2). The results suggest that EMS possesses potent anti-arthritis activity.

### EMS alleviated ankle joint histopathology in AA rats

**Figure 3.**
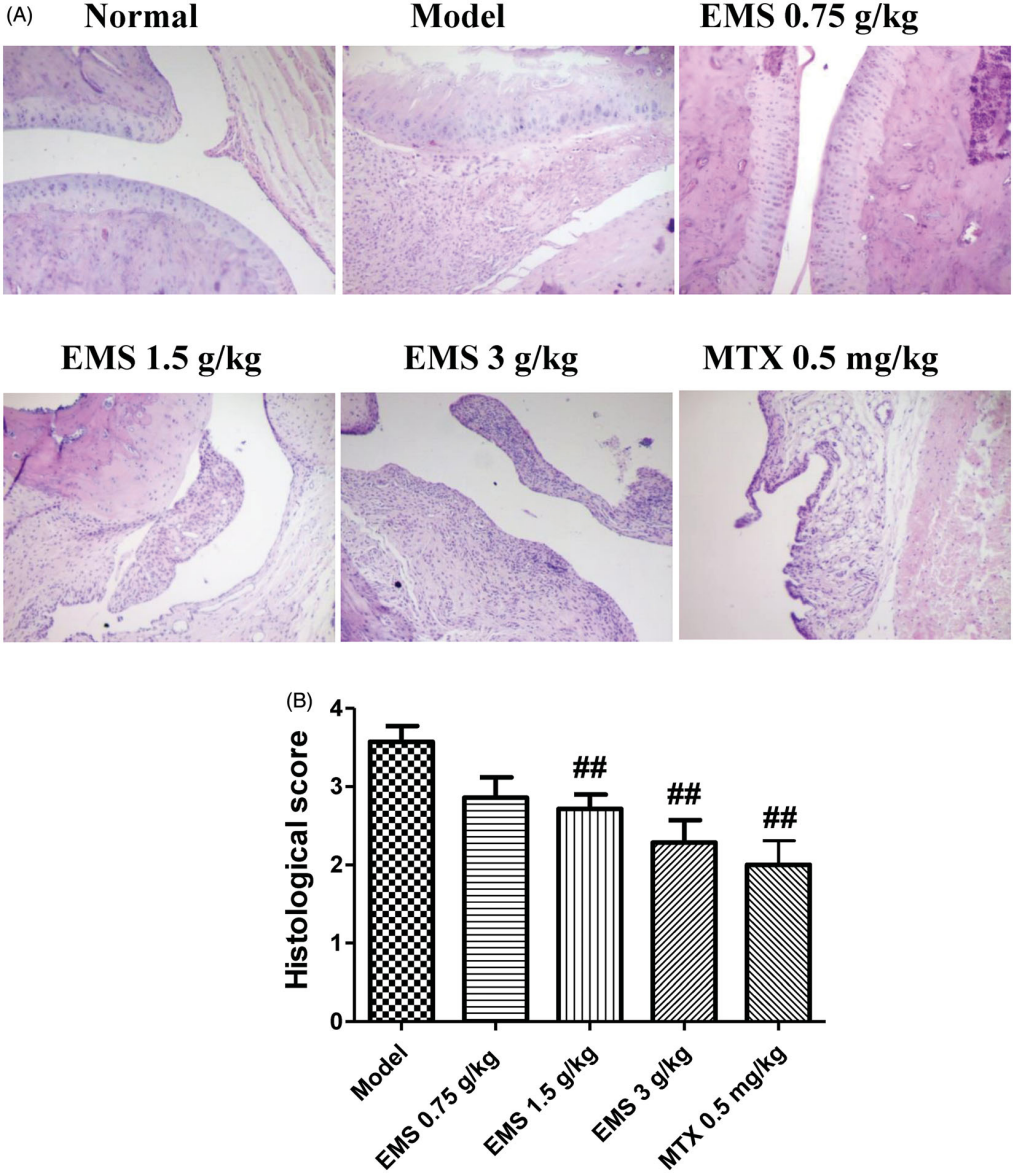
Effects of EMS on the joint histopathology of AA rats. (A) Representative images of joint histopathology are shown (HE, ×100). (B) Histological were scored as described in methods. Data are expressed as the mean ± SD for six animals in each group. ^##^*p*< 0.01 vs. model.

Histopathology is the most informative technique for understanding the manifestations of arthritic disease. Compared to normal rats, the ankle joint histopathology in AA rats was characterized by inflammatory cell infiltration, pannus formation, synovial proliferation, and erosion of bone and articular cartilage. These abnormal pathological changes were significantly alleviated in AA rats after EMS (1.5 and 3 g/kg) and MTX (0.5 mg/kg) administration (Figure 3).

### EMS inhibited FLS and T cell proliferation in AA rats

**Figure 4.**
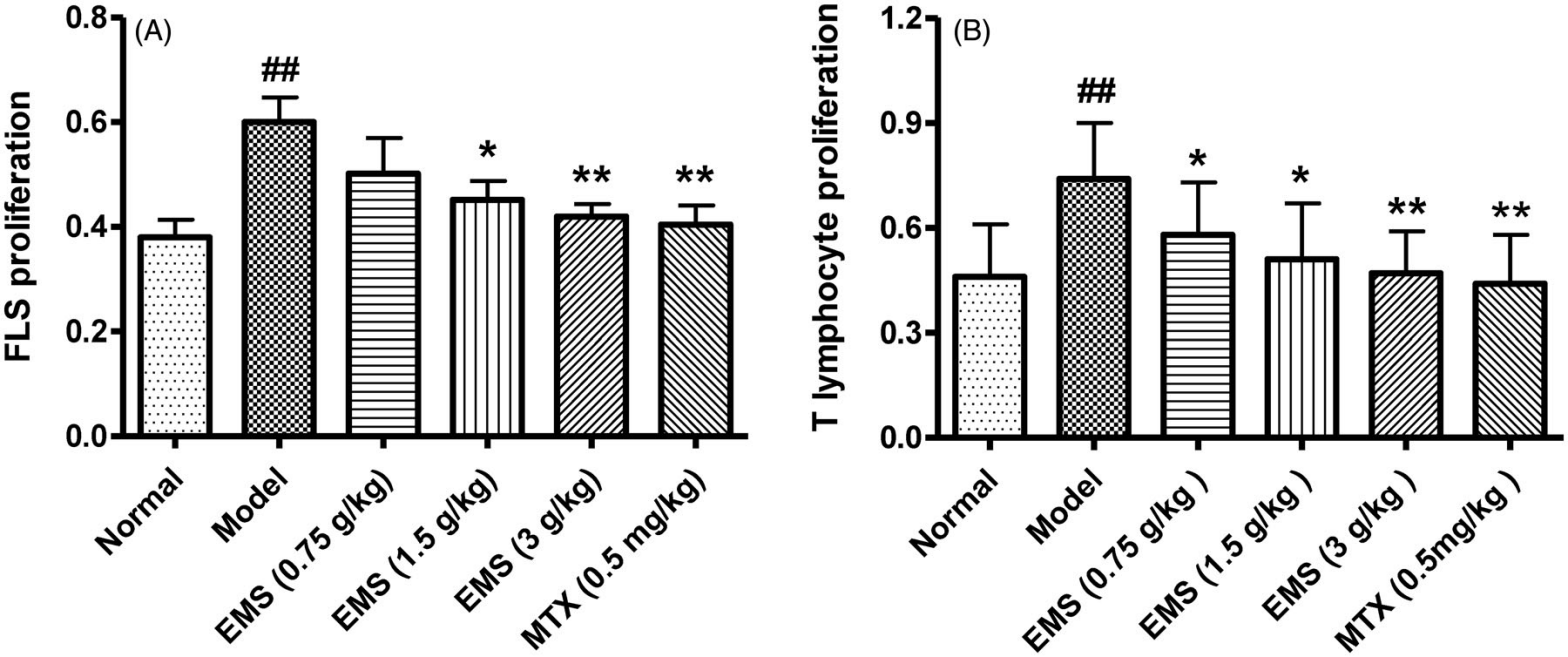
Effects of EMS on FLS and T cell proliferation in AA rats. (A) EMS inhibits FLS proliferation in AA rats. (B) EMS inhibits T cell proliferation in AA rats. ^##^*p*< 0.01 vs. normal, *p< 0.05, **p< 0.01 vs. model (mean ± SD, *n* = 6).

FLS is both an effector cell and a target cell. Abnormal FLS proliferation is a typical sign of RA. In AA rats, FLS proliferation was significantly increased compared to that in normal rats. Treatment with EMS significantly inhibited abnormal FLS proliferation in AA rats. In addition, Con A-induced T cell proliferation in AA rats was significantly increased compared to that in the normal group. EMS (0.75, 1.5 and 3 g/kg) and MTX (0.5 mg/kg) treatment also caused a reduction in T cell proliferation (Figure 4).

### EMS regulated the balance between Treg cells and Th17 cells of splenocytes in AA rats

**Figure 5.**
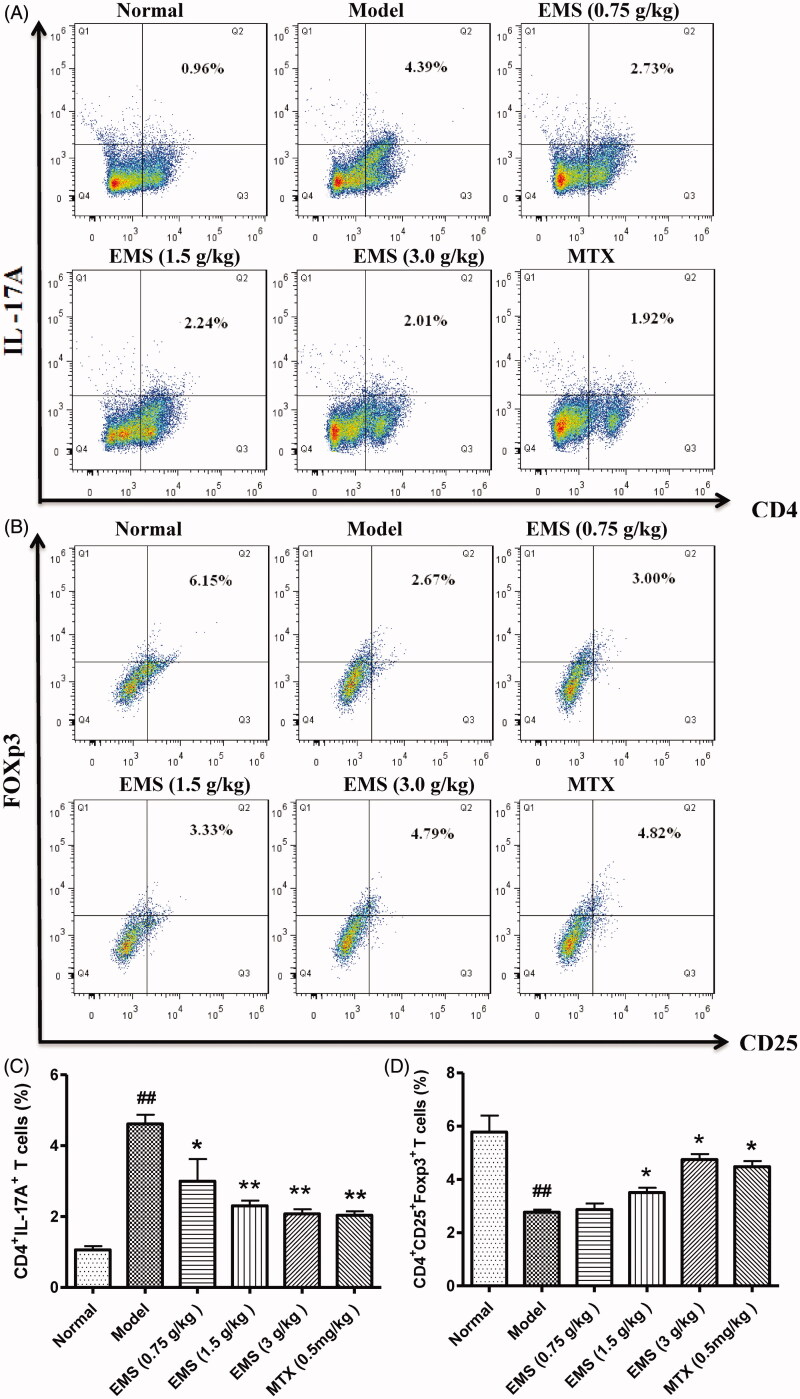
Effects of EMS on the proportion of Th17/Treg cells in AA rats. Splenocytes were obtained on day 32 and the proportion of CD4^+^IL-17A^+^ Th17 cells (A, C) and CD4^+^CD25^+^Foxp3^+^ Treg cells (B, D) were analysed by flow cytometry. Values are presented as mean ± SD of three animals per group. ^##^*p*< 0.01 vs. normal, *p< 0.05, **p< 0.01 vs. model.

To determine whether EMS can modulate the proportions of different T cells during AA, we investigated the profiles of Treg cells (CD4^+^CD25^+^Foxp3^+^) and Th17 cells (CD4^+^CD17^+^) in splenocytes via flow cytometry. EMS treatment was found to decrease the percentage of Th17 cells in splenocytes and to increase the percentage of Treg cells compared to the AA group (Figure 5(A,B)).

### EMS regulated the level of cytokines in serum of AA rats

**Figure 6.**
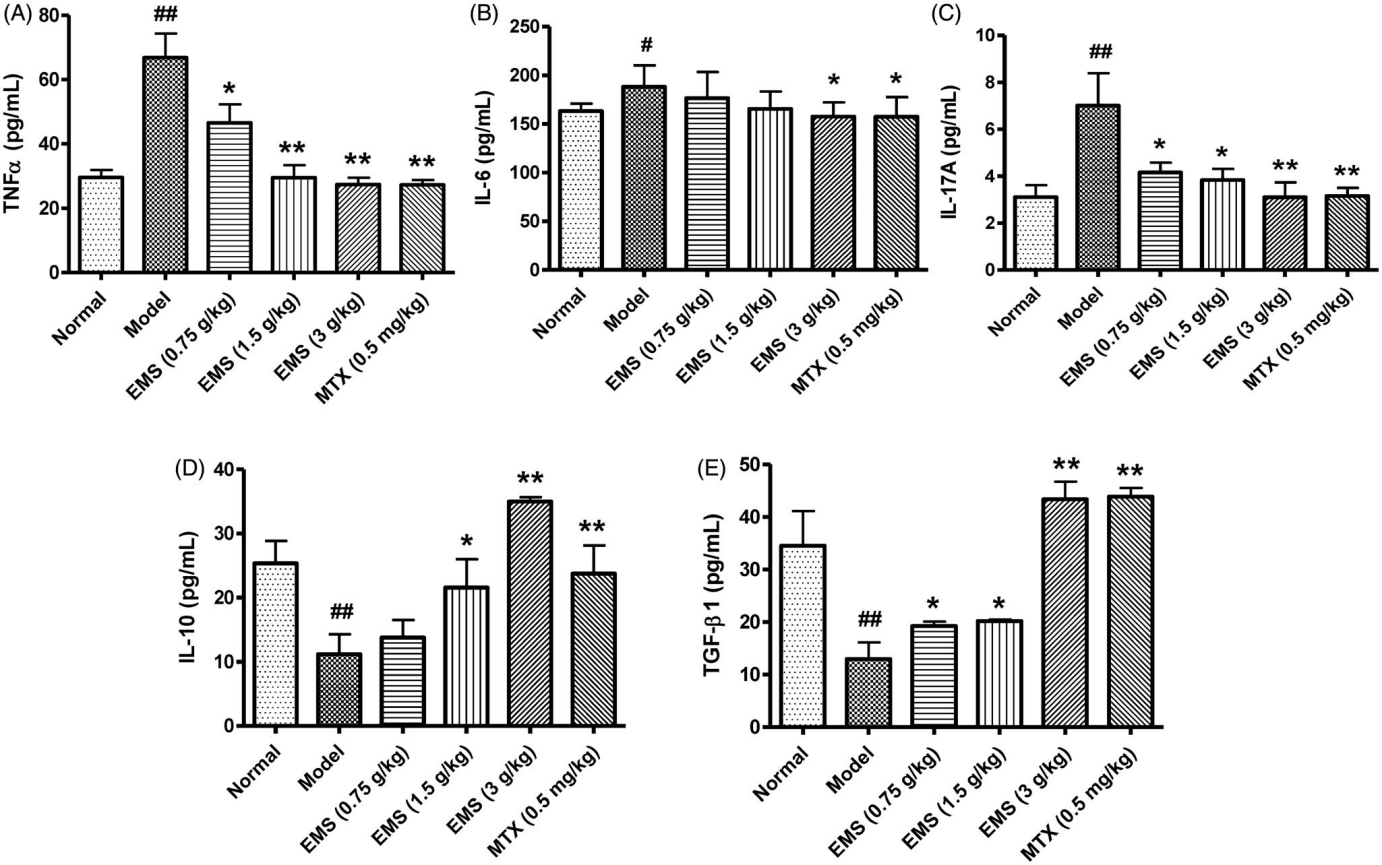
Effects of EMS on the level of cytokines in serum of AA rats. Cytokine levels in serum were detected by ELISA. (A) TNF-α, (B) IL-6, (C) IL-17A, (D) IL-10 and (E) TGF-β1. Values are presented as mean ± SD of eight animals per group. ^#^*p*< 0.05, ^##^*p*< 0.01 vs. normal, *p< 0.05, **p< 0.01 vs. model.

The levels of cytokines in serum were detected by ELISA. Levels of proinflammatory cytokines (IL-17A, TNF-α and IL-6) were increased in AA rats compared to those in normal rats, whereas the levels of anti-inflammatory cytokines (IL-10 and TGF-β1) were decreased (Figure 6(A–E)). After treatment with EMS and MTX, the levels of TNF-α, IL-6 and IL-17A were reduced, whereas those of the anti-inflammatory cytokines (IL-10 and TGF-β1) were significantly increased (Figure 5(A–E)). These results suggest that the ability of EMS to induce protection against AA involves the re-establishment of a balance between pro-inflammatory cytokines and anti-inflammatory cytokines.

## Discussion

RA is a chronic autoimmune disease involving various immune cells including dendritic cells, T cells and B cells. The most typical features of RA include FLS proliferation and cartilage and bone destruction of joints (Hu et al. 2019). Numerous studies have reported that an imbalance in Treg and Th17 cells plays a role in the pathogenesis of RA (Kosmaczewska et al. 2011; Alunno et al. 2015). Treg cells facilitate the maintenance of self-tolerance by producing anti-inflammatory cytokines such as IL-10 and TGF-β. Th17 cells which are abundant in the synovial fluid, can promote the migration, invasion and proliferation of FLS, the production of proinflammatory cytokines, and mediate inflammation and joint destruction in RA. Furthermore, the inflammatory cytokines, IL-6 and TNF-α, promote Th17 cell differentiation and block the differentiation and activity of Treg cells, thus increasing the ratio of Th17/Treg cells, which further aggravates RA (Assier et al. 2010). Therefore, restoration of the Th17/Treg balance and suppression of inflammatory cytokines may relieve the symptoms of RA (Tanaka et al. 2012).

AA is a classical animal model of RA in which the disease is mainly mediated by T cells. It is often used to study the underlying mechanisms of RA and the potential therapeutic effects of an intervention. Previous studies showed that EMS inhibited inflammatory events and iNOS expression by inhibiting MAPK activation and the NF-κB pathway (Chen et al. 2014). Furthermore, San Miao San, a related herbal formula, was found to display anti-inflammatory effects in an AA rat model (Lam et al. 2008). The present study demonstrated that EMS significantly reduced paw swelling and polyarthritis index and alleviated the progression of AA in rats. Histological evaluation showed that EMS treatment leads to a reduction in synovium hyperplasia and inflammatory cell infiltration, thereby confirming that EMS shows therapeutic and protective effects in the AA rat model.

To further elucidate the possible mechanisms underlying the therapeutic effects of EMS on AA, we sought to investigate the proliferation of FLS and levels of inflammatory cytokines. The results showed that EMS treatment both inhibited the proliferation of FLS, and reduced the level of TNF-α and IL-6 in serum.

The pathophysiology of RA is known to involve an immune dysfunction of T cells. Th17 cells are effector T cells that secrete proinflammatory cytokines, such as IL-17 and TNF-α. The actions of these proinflammatory cytokines are synergized in stimulating T cell proliferation and IL-6 production in synoviocytes. IL-6 production further increases Th17 differentiation (Zheng et al. 2014). Previous research has demonstrated that IL-17 is overexpressed in RA patients. Inhibiting the generation of Th17 cells and secretion of IL-17 cytokine can improve RA symptoms. However, Treg cells (CD4^+^CD25^+^Foxp3^+^) can also prevent autoimmune responses by secreting anti-inflammatory cytokines. IL-10 suppresses the expression of Th17 cells and promotes Treg cells (Heo et al. 2010). TGF-β1 plays an important role in Treg cell generation and expansion in peripheral tissues (Sun et al. 2019), which can prevent and protect against autoimmune arthritis (Astry et al. 2015; Lee et al. 2018).

To understand the role of T cells in AA in rats, we assessed T cell proliferation and levels of different T cell subtypes. The spleen was isolated and cells were processed for the detection of T cell subtypes by flow cytometry. IL-17A and Foxp3 represent the level of Th17 cells and Treg cells, respectively. In the AA model rats, the percentage of CD4^+^IL-17A^+^ Th17 cells and the levels of Th17-related proinflammatory cytokines were significantly elevated. With EMS treatment, there was an evident decrease in the proportion of CD4^+^IL-17A^+^ T cells and in the levels of pro-inflammatory cytokines (IL-17A, TNF-α and IL-6). In addition, EMS inhibited T cell proliferation, increased the expansion of the CD4^+^CD25^+^Foxp3^+^ Treg cell population, and inhibited disease progression. The levels of IL-10 and TGF-β1 in serum, two anti-inflammatory cytokines, also increased after EMS administration.

## Conclusions

EMS increased the percentage of Treg cells and the level of anti-inflammatory cytokines, and decreased the number of Th17 cells and the levels of proinflammatory cytokines, thereby improving disease symptoms and inhibiting the development and progression of AA in rats. These insights may help further develop EMS as a potent immunosuppressive agent and provide an experimental basis for the clinical treatment of RA.
